# The first mouse mutants of *D14Abb1e* (*Fam208a*) show that it is critical for early development

**DOI:** 10.1007/s00335-014-9516-0

**Published:** 2014-04-30

**Authors:** Sarah K. Harten, Timothy J. Bruxner, Vandhana Bharti, Marnie Blewitt, Thi-My-Tam Nguyen, Emma Whitelaw, Trevor Epp

**Affiliations:** 1 Epigenetics Laboratory, QIMR Berghofer Medical Research Institute, Herston, QLD 4006 Australia; 2 Molecular Medicine Division, The Walter and Eliza Hall Institute of Medical Research, University of Melbourne, Melbourne, VIC 3050 Australia; 3 Present Address: La Trobe Institute for Molecular Science, Department of Genetics, La Trobe University, Bundoora, VIC 3086 Australia; 4 Present Address: Institute of Molecular Genetics of the ASCR, v.v.i. Videnska, 1083 Prague 4 Czech Republic

## Abstract

An ENU mutagenesis screen to identify novel epigenetic modifiers was established in mice carrying a multi-copy GFP transgene, which is expressed in a variegated manner in erythrocytes and is highly sensitive to epigenetic silencing. The screen has produced mouse mutants of both known modifiers of epigenetic state, such as *Dnmt1* and *Smarca5*, and novel modifiers, such as *Smchd1* and *Rlf*. Here we report two mouse lines generated from the screen, *MommeD6* and *MommeD20*, with point mutations in *D14Abb1e*. These are the first mouse mutants of *D14Abb1e* (*also* *known* *as* *Fam208a*), a gene about which little is known. Heterozygous intercrosses show that homozygous mutants from both the *MommeD6* and *MommeD20* lines are not viable beyond gastrulation, demonstrating an important role for D14Abb1e in development. We demonstrate that haploinsufficiency for *D14Abb1e* effects transgene expression at the RNA level. Analysis of the predicted D14Abb1e protein sequence reveals that it contains putative nuclear localisation signals and a domain of unknown function, DUF3715. Our studies reveal that D14Abb1e is localised to the nucleus and is expressed in skin and testes.

## Introduction

**Fig. 1:**
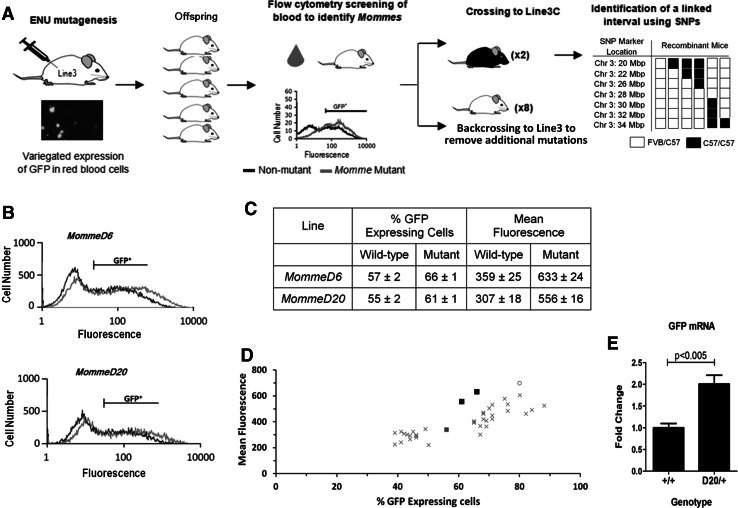
*MommeD6* and *MommeD20* are mutant lines identified from an ENU mutagenesis screen. **a** A schematic overview of the design of the screen. Briefly, male FVB/NJ mice carrying a multi-copy GFP transgene array (*Line3*), which is sensitive to epigenetic silencing, were injected with ENU, a chemical mutagen ENU and mated with female *Line3* mice. Offspring were screened, via flow cytometry, for changes in GFP expression using a drop of blood from the tail. Mice carrying mutations that caused a shift in the percentage of red blood cells expressing GFP were either (1) backcrossed to unmutagenised *Line3* mice to remove additional mutations or (2) backcrossed twice to C57BL/6J mice carrying the transgene (*Line3C*) to produce recombinant mice, which were used for linkage analysis. Causative mutations were identified by sequencing candidate genes from the linked interval. **b** Representative GFP expression profiles of wild-type and heterozygous mutant *MommeD6* and *MommeD20* mice, showing erythrocyte fluorescence (*x*-axis) versus the number of cells detected at each fluorescence level (*y*-axis). The GFP+ gate was set to exclude 99.9 % of wild-type cells, i.e. cells with no GFP transgene. Mice shown in each graph are from a single litter, since day to day variation in FACS readings prevents pooling of multiple litters (*n* = 3 mice for each FACS profile). The profiles of wild-type and mutant mice are shown in *black* and *red*, respectively. Data are representative of observations from over 100 litters per line. **c** Table showing representative percentage of GFP-expressing cells and mean fluorescence per expressing cell for *MommeD6* and *MommeD20* (mean ± SD). **d** A graph depicting Mean fluorescence versus percentage of GFP-expressing cells for wild-type *Line3* mice (blue square) and heterozygous mice from all *MommeD* lines reported to date, including *MommeD6* and *MommeD20* (*black* *squares*) and *MommeD7* (*grey* *circle*) mice. All other *MommeD* lines are shown as grey crosses. **e** Real-time RT-PCR analysis of GFP mRNA from spleens of age-matched wild-type and heterozygous *MommeD20* mutants, for *n* = 5 mice per genotype (Color figure online)

Epigenetic modifications of the DNA and the chromatin proteins that package it play critical roles in the establishment of normal patterns of gene expression in multi-cellular organisms (Feinberg 2007). Errors in this process can lead to defects in development and disease (Berdasco and Esteller 2013; Feil and Fraga 2011; Issa and Baylin 1996). To identify genes involved in the establishment and maintenance of the epigenetic state of the genome in mammals, our group designed an ENU (*N*-ethyl-*N*-nitrosourea) mutagenesis screen in mice (Blewitt et al. 2005). The screen is based on the observation that multi-copy transgene arrays are susceptible to silencing (Martin and Whitelaw 1996). The FVB/NJ mouse line used in our screen, *Line3*, carries a multi-copy transgene array, containing a green fluorescent protein (GFP) transgene under the control of the human alpha globin promoter and enhancer that is expressed in a variegated manner i.e. approximately 55 % of erythrocytes express GFP, whereas the remaining 45 % do not because the transgene is epigenetically silenced (Preis et al. 2003). Alleles that variegate in this way are known as metastable epialleles, and the percentage of expressing cells is sensitive to the levels of proteins involved in the establishment of epigenetic state (Rakyan et al. 2002). Wild-type male FVB/NJ mice carrying the GFP transgene (*Line3*) were injected with ENU to induce mutations in spermatogonial stem cells, and then mated with untreated *Line3* dams. Offspring were weaned at three weeks, at which time a drop of blood was collected for screening via flow cytometry. Offspring which showed a shift in the percentage of GFP-expressing cells were selected for heritability testing, by backcrossing to unmutagenised, wild-type (*Line3*) mice. If altered GFP expression was observed across multiple generations, the mutant mouse line was designated as a *MommeD* (*Modifier* *of* *murine* *metastable* *epiallele* *Dominant*) (Fig. 1a) (Blewitt et al. 2005).

To date the screen has produced around 40 *MommeD* mouse lines and causative mutations have been identified in 29 of these (Daxinger et al. 2013). The finding of several well-studied epigenetic modifiers, such as DNA methyltransferases, histone modifying enzymes and chromatin remodelers, validates the design of the screen (Ashe et al. 2008; Chong et al. 2007; Daxinger et al. 2013; Youngson et al. 2013). This report focuses on two additional mouse lines, *MommeD6* and *MommeD20*, which we have now found to contain mutations in *D14Abb1e* (DNA segment, Chr 14, Abbott 1 expressed). *MommeD6* and *MommeD20* are the first mouse mutants to be described for *D14Abb1e*.

## Materials and methods

### Mouse lines

The ENU mutagenesis screen was conducted as described previously (Blewitt et al. 2005). *Line3* and *Line3C* mice are homozygous for a multi-copy GFP transgene under the control of the human alpha globin promoter and linked to the HS-40 enhancer and are inbred on the FVB/NJ and C57BL/6J lines, respectively. Inbred FVB/NJ and C57BL/6J mice were originally purchased from ARC Perth (Perth, WA, Australia). *MommeD6* and *MommeD20* mice used in this study were backcrossed to unmutagenised *Line3* for at least five generations to remove additional ENU mutations from the genome. Sperm from *MommeD6* and *MommeD20* mutant mouse lines has been cryopreserved and is available via the Australian Phenome Facility (http://apf.anu.edu.au). All animal work was approved by the QIMR Berghofer Animal Ethics Committee.

### Flow cytometry

Mice were weaned at three weeks of age and a drop of blood was collected into a tube of Osmosol buffer (Lab Aids Pty Ltd., Narrabeen, NSW, Australia). Samples were analysed on a FACScan (Becton Dickinson, Franklin Lake, NJ, USA). A Gate was set so as to demarcate GFP-expressing cells, such that 99.9 % of wild-type erythrocytes were excluded. Prior to identification of the causative mutation, mice were classified as phenotypic wild-types or phenotypic mutants on the basis of their FACS profile.

### Linkage analysis

Mutants were backcrossed to *Line3C* for two generations to generate C57BL/6J/FVB/NJ N2 recombinants. PCR primers were used to amplify regions containing either microsatellite or single nucleotide polymorphisms (SNPs) which differed between C57BL/6J and FVB/NJ strains. SNP markers were chosen which result in the creation or destruction of a restriction enzyme digest site. PCR products were resolved on agarose gels. Within the linked interval wild-type mice will display a C57BL/6J/C57BL/6J genotype, whereas mutant mice will be C57BL/6J/FVB/NJ. Linked interval coordinates refer to the NCBI37/mm9 genome assembly. Primer sequences are available on request.

### Genotyping

Mouse tissue (either a 0.5 cm piece of tail or embryo tissue) was digested with tail lysis buffer containing 1 mg/mL Proteinase K (Astral Scientific, Australia) overnight at 55 °C. Samples were heated to 95 °C for 5 mins to inactivate Proteinase K. PCRs of an interval containing the mutation were performed with the following primers *MommeD6*: For-GGCCTTTGGTCAGAAAACCT Rev-GTTAAAATATGTTACTGATGATGGCTCA; *MommeD20*: For- GCCTTTTAGGCGGAGTTTTC Rev- GAAACGCTTCAAACCTGAGC. Genotypes of *MommeD20* samples were determined by Sanger sequencing, using Big Dye 3.1 (Applied Biosystems, Foster City, CA). *MommeD6* samples were genotyped either by Sanger sequencing or restriction enzyme digest using AciI (New England Biolabs, Beverly, MA). The *MommeD6* mutation results in the creation of an AciI site.

### Cell culture and siRNA

HeLa cells were maintained in complete media i.e. DMEM supplemented with 10 % foetal calf serum and penicillin/streptomycin. All culture reagents were purchased from Life Technologies (Gaithersburg, MD). For siRNA transfection experiments, cells were plated in complete media 24 h before transfection. Immediately prior to transfection, media were changed to DMEM only. Cells were transfected with Lipofectamine 2000 (Life Technologies, Gaithersburg, MD) according to the manufacturers’ instructions. Serum was added after 4 h to give a final concentration of 10 %. Cells were harvested after 36 hrs for Western blotting. The following siRNAs were obtained from Qiagen: All Stars Negative Control, Hs_C3orf63_7: GAGGAACTTAGTACTCCAGAA and Hs_C3orf63_4: TTCCGAGTTCATATATTCTAA

### Real-time RT-PCR and splice site analysis

RNA was extracted using Trizol (Life Technologies, Gaithersburg, MD) according to the manufacturer’s instructions. cDNA was prepared using 1st strand cDNA synthesis kit (Life Technologies, Gaithersburg, MD). Real-time PCR was performed on a Rotor-Gene 6000 (Corbett Research, Mortlake, NSW, Australia) using Sybr Green (Life Technologies, Gaithersburg, MD). Primers: GFP For: CTACCCCGACCACATGAAGC and GFP Rev: CTTGTAGTTGCGTCGTCCT. All values were normalised to the house-keeping gene β-actin (β-actin For: CCAGAGCAAGAGAGGTATC and β-actin Rev: GACCAGAGGCATACAGGGAC). For splice site analysis a forward primer from exon 1 and a reverse primer from exon 4 were used to amplify cDNA from both wild-type and *MommeD20* mutants. Products were separated on an agarose gel and individual bands were excised from the gel and purified using a gel extraction kit (Qiagen) according to the manufacturer’s instructions. Products were sequenced as described above.

### Western blotting

Protein lysates were prepared using an 8 M urea buffer and quantified using a BCA assay (Thermo Fisher Scientific, Waltham, MA), according to the manufacturer’s instructions. Lysates were separated on pre-cast TGX gels (Bio-Rad Laboratories, Richmond, CA) and transferred onto Immobilon PVDF membranes (Millipore, Bedford, MA). Membranes were blocked for 20 mins in 5 % milk, 1 % BSA solution in PBST before incubating overnight at 4 °C with the appropriate primary antibody. Antibodies used were anti-FAM208A (SC99819, Santa Cruz Biotechnology, Santa Cruz, CA), anti-FAM208A (HPA017142, Sigma-Aldrich, St Louis, MO), anti-GAPDH (Cell Signaling Technology, Beverly, MA), anti-Smarca5 (Abcam, Cambridge, UK) and anti-γ-tubulin (Sigma-Aldrich, St Louis, MO). The following day, membranes were washed with PBST, incubated with the appropriate secondary antibody at room temperature for 1 hour (Dako Corp, Carpinteria, CA) and then washed again with PBST. Membranes were developed using ECL (Bio-Rad Laboratories, Richmond, CA) and images captured using a digital chemiluminescent detection system (DNR Bio-Imaging Systems Ltd, Jerusalem, Israel).

### Histology

Embedding and sectioning of tissue, and H&E staining of sections was performed by the QIMR Berghofer Histology Service. Slides were scanned using an Aperio slide scanner (Aperio Technology, Vista, CA).

### Statistical analyses

Statistical significance of quantitative data was determined by two-tailed Student’s *t* test. The proportions of genotypes were compared to expected Mendelian ratios using a *χ* ^2^ test. For all datasets a minimum of three biological replicates were analysed.

## Results

### *MommeD6* and *MommeD20* showed an increase in the percentage of red blood cells expressing the GFP transgene and higher mean fluorescence per expressing cell

In our initial description of the screen we reported the FACS profiles for the first six mutants identified from the screen, including *MommeD6* (Blewitt et al. 2005). *MommeD6* ^+/−^ mice showed an increase in the percentage of red blood cells expressing GFP compared to wild-type littermates. We also noted that *MommeD6* ^+/−^ displayed a higher mean fluorescence level per expressing cell than the three other mutant lines with an increased percentage of GFP-expressing cells. Since then we have expanded the screen and we now report a new mutant line, *MommeD20*, which displays a similar shift in both the percentage of GFP-expressing cells and mean fluorescence per expressing cell (Fig. 1c). Representative FACS profiles for both *MommeD6* and *MommeD20* are shown in Fig. 1b. Graphing the percentage of GFP-expressing cells *vs* mean fluorescence for all *MommeD* lines revealed that *MommeD6* ^+/−^ and *MommeD20* ^+/−^ had markedly higher mean fluorescence per expressing cell, relative to the observed increase in the percent of cells expressing GFP, than other lines identified in the screen (Fig. 1d). A third line, *MommeD7*, also displayed high mean fluorescence, as was previously noted (Ashe et al. 2008). Analysis of *MommeD7* ^+/−^ blood samples revealed a high reticulocyte count and a mutation was subsequently identified in the canonical polyadenylation signal of the haemoglobin, beta major gene (Brown et al. 2013). Full blood count analysis and reticulocyte counts of *MommeD6* ^+/−^ mice showed no differences from wild-type littermates (data not shown). We determined the level of GFP mRNA in *MommeD20* ^+/−^ spleen using real-time RT-PCR. Significantly higher levels of GFP mRNA were observed in *MommeD20* ^+/−^ mice compared to wild-types (Fig. 1e).

### *MommeD6* and *MommeD20* contain mutations in *D14Abb1e*

**Fig. 2:**
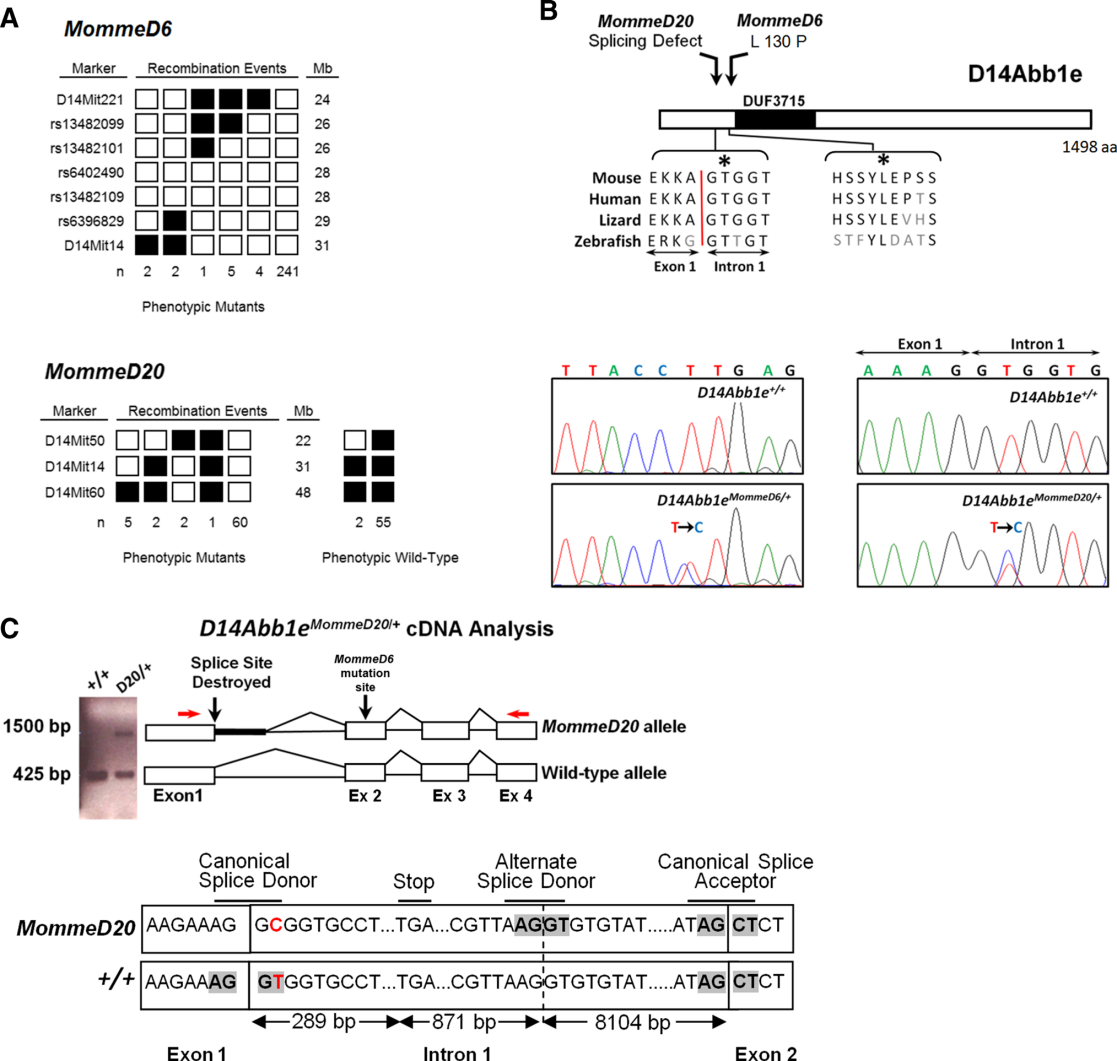
*MommeD6* and *MommeD20* contain mutations in *D14Abb1e*. **a** Mapping interval for *MommeD6* and *MommeD20* mice. *MommeD6* and *MommeD20* mutants were produced in the FVB/NJ strain and backcrossed to C57BL/6J mice to produce recombinant mice. The results for markers used around the linked interval are shown. *Left* margin, markers used; *right* margin, marker positions on mouse chromosome 14 (NCBI Build 37, August 2008). *Filled* *boxes* represent markers homozygous for the C57BL/6J allele; *open* *boxes* represent markers heterozygous for FVB/NJ and C57BL/6J. Data represent defining recombinant events, with the number of mice identified with each genotype below. An interval of 2.5 Mb was defined for *MommeD6*, between rs13482101 and rs6396829. An interval of 9 Mb was defined for *MommeD20*, between D14Mit50 and D14Mit14. **b** Representative DNA sequence electropherograms for wild-type, *MommeD6* and *MommeD20* mutant mice. In both *MommeD6* and *MommeD20*, a single base pair mutation was detected in *D14Abb1e* (*MommeD6* Exon 2, T → C; *MommeD20* Intron 1, T → C). **c** cDNA analysis of the exon 1–exon2 region of *D14Abb1e* in wild-type and *MommeD20* mutant mice. *Red* *arrows* show location of primer sites used to amplify cDNA from wild-type and *D14Abb1e* ^*MommeD20/+*^ mice. Amplified products are shown on a representative image of an agarose gel. Schematic diagrams show splicing of D14Abb1e, based on Sanger sequencing of bands detected on gel. The T → C point mutation found in *MommeD20* mice, shown in *red*, occurs within the slice donor site of intron 1. Sequencing of the ~1,500 bp band, detected only in mutant samples, showed partial incorporation intron 1, including a predicted premature stop codon

To determine the causative mutations, each line was backcrossed twice to C57BL/6J mice carrying the GFP transgene (*Line3C*), to produce NF2 recombinant mice. These recombinants were used to map the location of the causative mutation using traditional SNP and microsatellite markers. *MommeD6* mapped to a 2.5 Mbp interval on Chromosome 14 between the markers rs13482101 (26,285,297 bp) and rs6396829 (28,814,048 bp) (Fig. 2a). An overlapping linked interval was identified in *MommeD20* (Chromosome 14: 22–31 Mbp). This interval fits with all of the phenotypic wild-type mice analysed (57/57) and all but one of the phenotypic mutant mice analysed (69/70). All but one exon, including exon/intron junctions and splice sites, were sequenced for seven genes from the interval including; the homeobox genes *Cphx1* (cytoplasmic polyadenylated homeobox 1), *Duxbl1* (double homeobox B-like 1) and *Hesx1* (homeobox gene expressed in ES cells), SNORA71D and SNORA67 which encode untranslated, small nucleolar RNAs (snoRNAs), *Appl1* (adaptor protein, phosphotyrosine interaction, PH domain and leucine zipper containing 1), which has links to the nucleosomal remodelling and histone deacetylase (NuRD) complex (Banach-Orlowska et al. 2009; Feng and Zhang 2003; Miaczynska et al. 2004) and *D14Abb1e* (Chambers and Abbott 1996). A single mutation was identified in exon 2 of *D14Abb1e*, a T to C mutation, encoding a non-conservative amino acid change (L130P) at a highly conserved leucine residue (Fig. 2b). We have designated this mutant allele as *D14Abb1e* ^*MommeD6*^.

Candidate gene sequencing of *D14Abb1e* in *MommeD20* mutant mice revealed a T to C mutation at the canonical splice site exon1/intron1 (Fig. 2B). Analysis of cDNA prepared from wild-type and *MommeD20* heterozygous mutants was performed using primers designed to amplify across intron 1 of *D14Abb1e*. Wild-type cDNA produced a single PCR product of the expected size; however, a second, higher molecular weight product was also detected in *MommeD20* heterozygous samples. Sanger sequencing of the bands showed that splicing of the mutant allele was altered, resulting in the incorporation of additional 1,160 nucleotides. Translation of the abnormal mRNA species is predicted to result in the incorporation of 79 amino acids, of no substantial homology to any known protein, followed by a premature stop codon, which would elicit nonsense-mediated decay (Fig. 2c). We have designated this allele as *D14Abb1e* ^*MommeD20*^.

Almost nothing is known about *D14Abb1e* and, to our knowledge, *MommeD6* and *MommeD20* are the first reported mouse mutants. *D14Abb1e* shows evolutionary conservation within vertebrates. FAM208A, the human homologue of D14Abb1e, shows 82 % conservation. Analysis of the D14Abb1e amino acid sequence predicts a single predicted domain of unknown function (DUF3715) (Punta et al. 2012). Identification of *D14Abb1e* mutations in two independent mutant lines, *MommeD6* and *MommeD20*, provides strong support that these are the causative mutations.

### D14Abb1e protein is expressed in skin and testis

**Fig. 3:**
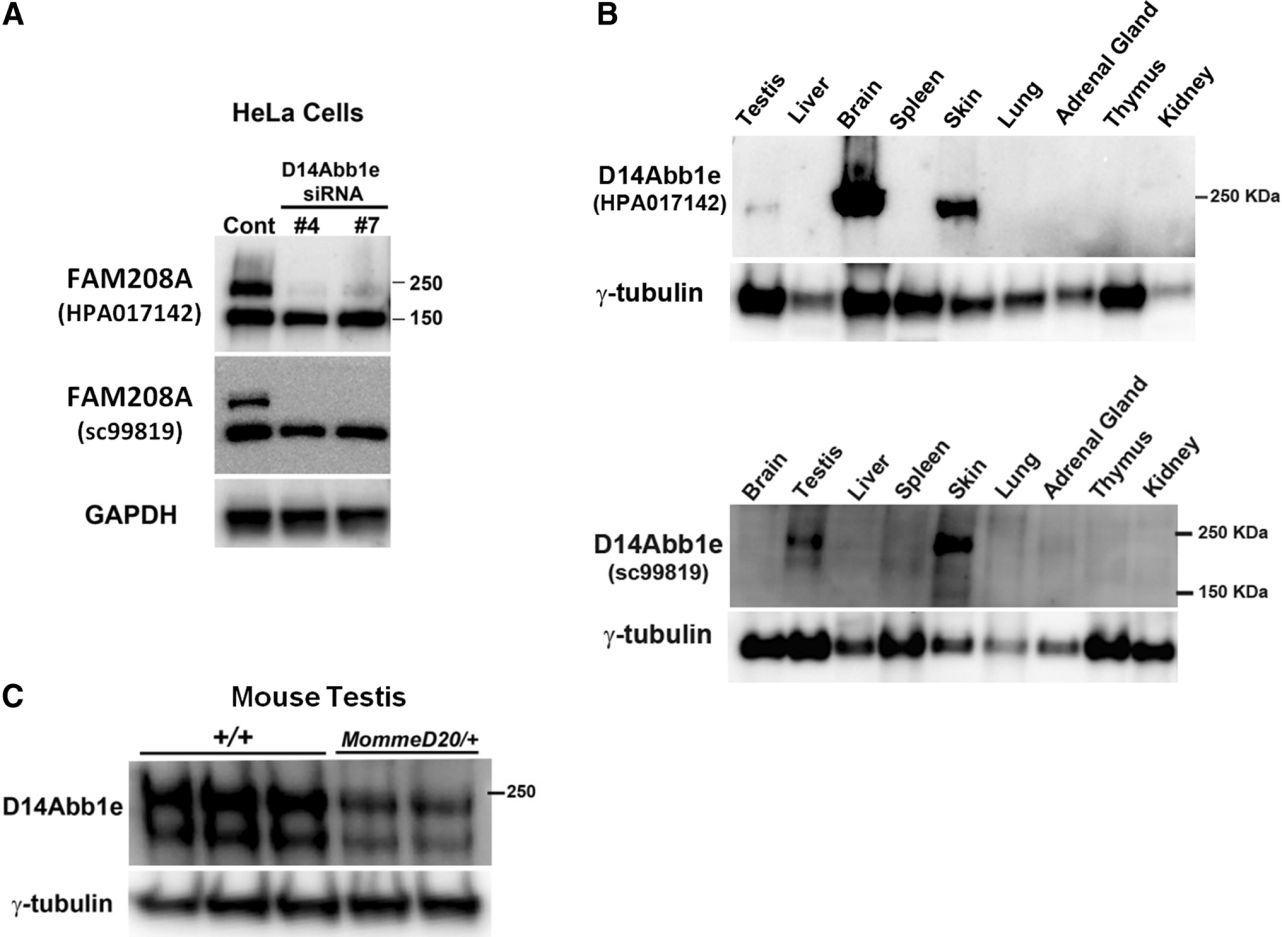
D14Abb1e protein is most highly expressed in skin and testis. **a** Representative Western blots of human (HeLa) protein lysates treated with siRNAs targeting FAM208A. Two anti-FAM208A antibodies which recognise independent epitopes, HPA017142 and sc99819, were used to probe duplicate Western membranes. Both membranes were also probed with an anti-GAPDH antibody. The GAPDH data presented correspond to the membrane blotted with HPA017142. **b** Representative Western blots showing expression of D14Abb1e in a panel of adult mouse tissues, blotted with two independent antibodies (HPA017142 and sc99819). γ-Tubulin was also blotted as a loading control. **c** Western blotting of D14Abb1e in protein lysates from wild-type and *D14Abb1e* ^*MommeD20/+*^ testis. A representative blot is shown. All Western analyses were performed with at least three independent sets of biological replicates. Representative Westerns are shown in each case

FAM208A is predicted to encode three protein variants in humans, with molecular weights of 140, 171 and 182 kDa (Artimo et al. 2012). In contrast, mouse D14Abb1e is predicted to encode a single isoform of 169 kDa (Artimo et al. 2012). To determine the ability of available antibodies to detect FAM208A, we treated HeLa cells with either a control siRNA or two independent siRNAs targeting FAM208A. Western blotting of control-treated cells revealed two bands, which were detected with two independent anti-FAM208A antibodies. The higher molecular weight band, whose size is consistent with the larger predicted isoforms of FAM208A, was reduced upon siRNA treatment; however, levels of the lower molecular weight band were unchanged (Fig. 3a). Both FAM208A siRNAs target a region common to the transcripts of all predicted FAM208A protein-coding variants. Additional non-coding FAM208A transcripts are predicted. Therefore, the band at 150 kDa may represent either a non-specific band or FAM208A variant not present in current prediction databases and not targeted by the siRNAs.

Next, we asked in which tissues D14Abb1e is most highly expressed by blotting a panel of protein lysates prepared from adult mouse tissues. Using an anti-D14Abb1e designed against a peptide from the C-terminal region of D14Abb1e (HPA017142) we observed strong signals in skin, brain and testis (Fig. 3b). Signals at the same molecular weights were observed in skin and testis samples blotted with a second anti-D14Abb1e antibody, (sc99819), designed against an independent epitope in the C-terminus of D14Abb1e, however no band was observed in brain lysates (Fig. 3b). Previous studies have detected D14Abb1e transcript in the brain (Chambers and Abbott 1996), but these findings suggest that further studies are needed to clarify the level of protein expression in this tissue (see ‘Discussion’). The *D14Abb1e* transcript produced by the *MommeD20* mutant allele is predicted to result in the incorporation of an early stop codon. To test the effect of the *MommeD20* allele on D14Abb1e protein expression we asked whether the signal was reduced in lysates from *D14Abb1e* ^*MommeD20/+*^ compared to wild-type mice. Western blots of testis lysates revealed less D14Abb1e in *D14Abb1e* ^*MommeD20/+*^ lysates compared to wild-types, as expected (Fig. 3c).

### D14Abb1e is localised to the nucleus

**Fig. 4:**
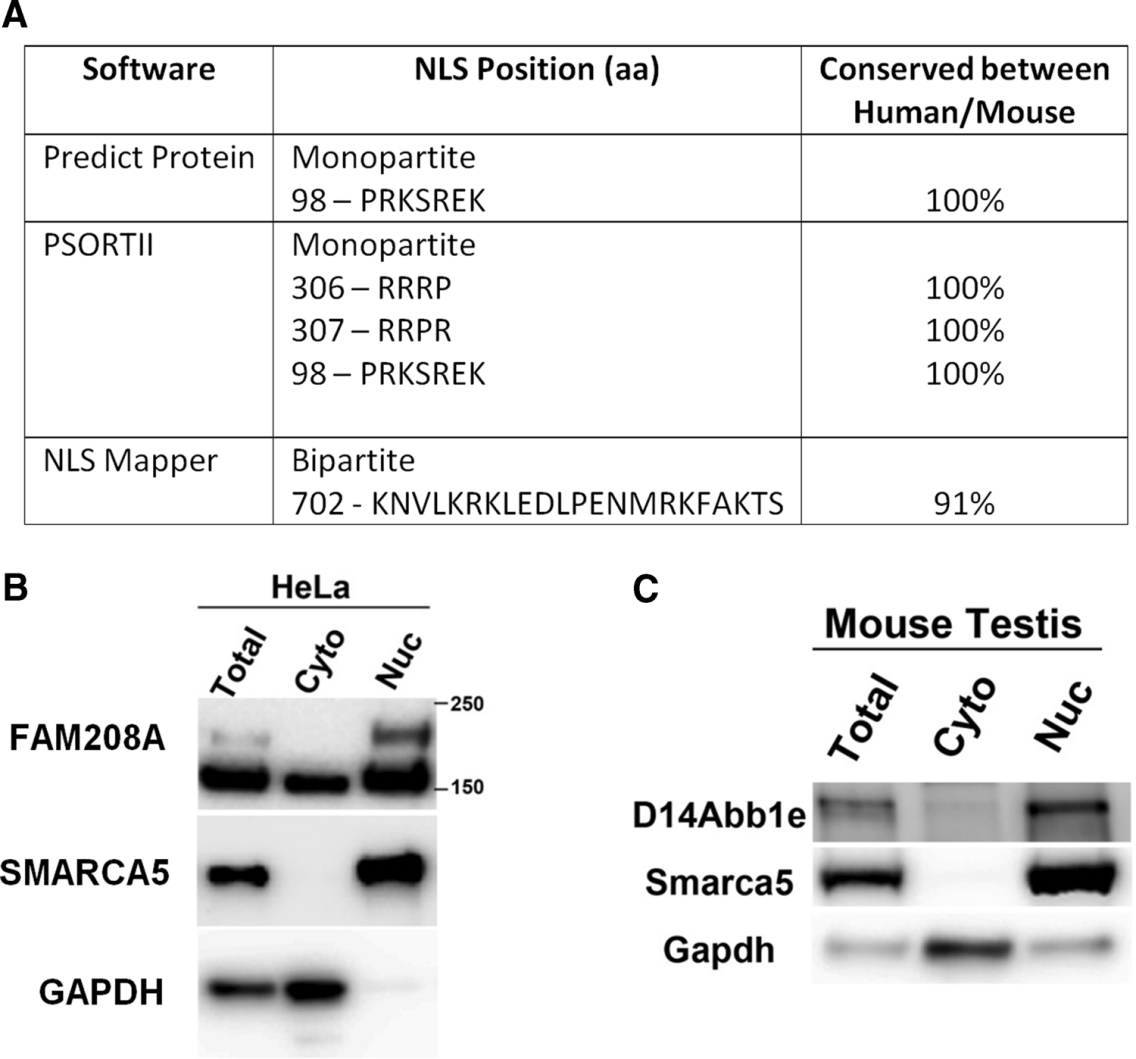
D14Abb1e is localised to the nucleus. **a** Bioinformatic analysis of the 1498 amino acid sequence of D14Abb1e performed using protein domain prediction programs Predict Protein, PSORTII and NLS Mapper, indicated the presence of multiple putative highly conserved nuclear localisation signals. **b**, **c** Western blots of total, nuclear and cytoplasmic cell fractions showing protein localisation of FAM208A/D14Abb1e in a human cell line (**b**) or mouse testis (**c**). Lysates were also blotted for Gapdh and Smarca5, which are known to be localised to the cytoplasm and nucleus, respectively. Data shown are representative of three independent experiments

Analysis of the primary amino acid sequence of FAM208A and D14Abb1e revealed the presence of monopartite and bipartite putative nuclear localisation signals that are conserved between species (Fig. 4a). Total, cytoplasmic and nuclear fractions were prepared from both human cell line (HeLa) and mouse testis and tested for the presence of the protein by Western analysis. In each case the protein was found to be localised in the nucleus (Fig. 4b, c and data not shown).

### D14Abb1e is critical for normal development

**Fig. 5:**
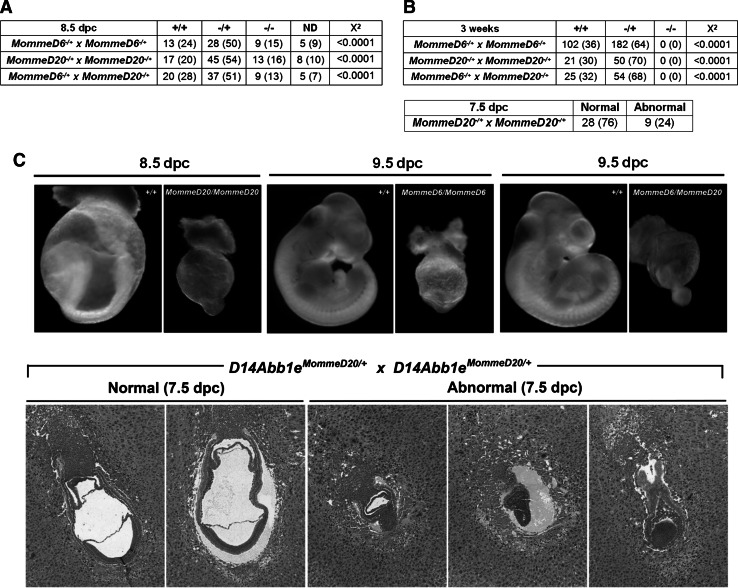
D14Abb1e is critical for normal development. **a** Genotypes of viable offspring from heterozygous intercrosses of *D14Abb1e* ^*MommeD6/+*^ or *D14Abb1e* ^*MommeD20/+*^ at 3 weeks of age. The results of compound *D14Abb1e* ^*MommeD6*^^/+^ × *D14Abb1e* ^*MommeD20/+*^ crosses is also shown. Data show the number of mice observed (and in brackets the percentage). The proportions of genotypes observed were compared to expected Mendelian ratios using the *χ* ^2^ test. **b** Timed matings at 8.5 and 7.5 dpc of *D14Abb1e* ^*MommeD6/+*^ or *D14Abb1e* ^*MommeD20/+*^ heterozygous intercrosses, or compound *D14Abb1e* ^*MommeD6/+*^ × *D14Abb1e* ^*MommeD20/+*^ crosses. Embryos from 8.5 dpc crosses were genotyped. Data show the number of mice observed (and in brackets the percentage). The proportions of genotypes observed, of normal developmental stage, were compared to expected Mendelian ratios using the *χ* ^2^ test. Histological examination of H&E sections was used to classify 7.5 dpc embryos as either developmentally normal or abnormal. **c** Representative photomicrographs of dissected embryos (8.5 and 9.5 dpc) and H&E sections from 7.5 dpc *D14Abb1e* ^*MommeD20/+*^ heterozygous intercrosses

*MommeD6* and *MommeD20* heterozygous intercrosses were established to examine viability of *D14Abb1e* mutant mice. Genotyping of offspring revealed that both intercrosses failed to produce any viable homozygous mutant offspring at three weeks of age (Fig. 5a). These results are consistent with our previous report of *MommeD6* intercrosses, prior to identification of the underlying mutation, in which we found no phenotypic homozygous offspring at weaning on the basis of FACS profiles (Blewitt et al. 2005). In each case approximately double the number of heterozygous offspring were observed compared to wild-types (*MommeD6* intercross: 102 wild-types and 182 heterozygotes; *MommeD20* intercross: 21 wild-types and 50 heterozygotes), suggesting that both the *MommeD6* and *Mommed20* alleles are semi-dominant and homozygous lethal. *D14Abb1e* ^*MommeD6/+*^ mice were also crossed to *D14Abb1e* ^*MommeD20/+*^ mice to test for genetic complementation. No compound heterozygous animals, *D14Abb1e* ^*MommeD6/MommeD20*^, out of 79 offspring, were observed at three weeks of age, as expected.

Timed matings of *MommeD6* and *MommeD20* intercrosses were performed to determine the stage of development at which *D14Abb1e* homozygous mutants fail. Characterisation of the *MommeD6* line, prior to the identification of the causative mutation, revealed arrest prior to 10.5 dpc (Ashe et al. 2008). Here we show that at 8.5 dpc, a mixture of developmentally normal and developmentally delayed embryos, were present (Fig. 5b). Empty decidua were also observed. Where possible, embryo tissue was collected and genotyped. Empty decidua or embryonic tissue that failed to genotype was recorded as not determined (ND). In both lines developmentally normal embryos were found to be wild-type or heterozygous mutants. Homozygous *MommeD6* and *MommeD20* mutants showed severe developmental delay and were present in less than expected numbers (Fig. 5b). Timed matings of *D14Abb1e* ^*MommeD6/+*^ mice crossed to *D14Abb1e* ^*MommeD20/+*^ mice, revealed that development of compound heterozygotes was also impaired, showing a similar gross morphology to *MommeD6* and *MommeD20* homozygotes (Fig. 5b). Representative pictures of wild-type, homozygous and compound heterozygous mutant embryos are shown in Fig. 5c. To examine the phenotype further, *D14Abb1e* ^*MommeD20/+*^ intercrosses were set up and dissections carried out at 7.5 dpc. Haemotoxylin and eosin staining was carried out on sections from 37 embryos. 28 were classified as developmentally normal and 9 were developmentally abnormal, showing failure to gastrulate. These embryos were not genotyped. Representative pictures are shown in Fig. 5c. These findings suggest that D14Abb1e is necessary for successful gastrulation.

## Discussion

This study describes two mouse mutants produced in our mutagenesis screen, *MommeD6* and *MommeD20*, which each carry a unique mutation in *D14Abb1e*. Identification of *D14Abb1e* mutations in two independent lines provides strong support that these are the causative mutations. These findings are also consistent with genetic complementation studies, which suggested disruption of a common gene or pathway, and with FACS profile data. The mutations in both *MommeD6* (non-conservative amino acid change) and *MommeD20* (splice site mutation) are expected to disrupt D14Abb1e function. Western blotting of lysates from *MommeD20* heterozygotes showed a reduction in D14Abb1e protein, consistent with it acting as a null allele. *MommeD6* and *MommeD20* have similar phenotypes, suggesting that the L130P mutation also creates a null allele.

Loss of *D14Abb1e* is critical for development, with homozygous mutants unable to progress beyond gastrulation. Further study is needed to uncover the molecular function of D14Abb1e in early development. Unlike most other mouse mutants produced from the screen, both *D14Abb1e* mutant lines show a substantial shift in mean fluorescence per expressing cell. Although the screen was designed to detect modifiers of transgene silencing, it is important to be aware that mutants may show a shift in their GFP FACS profile for other reasons. For example, mutations in genes alter the red blood cell count or affect stability of the GFP RNA or protein (Garneau et al. 2007). For instance, the high mean fluorescence per expressing cell observed in *MommeD7* results from a mutation in the polyadenylation signal of the haemoglobin, beta adult major chain gene which leads to low mean corpuscular volume and reticulocytosis (Brown et al. 2013). *MommeR1*, a mutant line identified from a modified screen to identify recessive mutants, also displays an unusually high mean fluorescence per expressing cell compared to shift in the percentage of GFP expressing cells. *MommeR1* was found to have a mutation in *Foxo3A* (Youngson et al. 2011). At this stage it is unclear whether either *Foxo3A* or *D14Abb1e* have a role in the establishment or maintenance of chromatin. Up-regulation of GFP mRNA levels in *D14Abb1e* mutant lines, combined with its nuclear localisation, is consistent with a role for D14Abb1e in regulating gene expression at the level of RNA, either by influencing transcription, potentially via an epigenetic mechanism, or via effects on post-transcriptional mechanisms of RNA regulation, for example splicing, polyadenylation, mRNA trafficking or mRNA decay (Di Liegro et al. 2014; Garneau et al. 2007).

D14Abb1e was initially isolated from a mouse cDNA library prepared from brain (Chambers and Abbott 1996); however, little is currently known about its molecular function. Our Western blot analysis of adult mouse tissues, performed with an anti-D14Abb1e antibody (HPA017142; C-terminal epitope), detected signal in skin, testes and brain tissue lysates. Using an independent antibody (sc99819; C-terminal epitope), we confirmed that the bands observed in skin and testes are likely to be D14Abb1e. However, despite the observed similar molecular weights in all three tissues, the latter antibody failed to detect D14Abb1e in brain lysates. It is possible that the HPA017142 antibody recognises a D14Abb1e isoform, which is expressed in brain and does not contain the epitope recognised by the sc99819 antibody. Further study is needed to examine the expression of D14Abb1e isoforms across different organs, both during development and in adult tissues.

D14Abb1e has also been isolated from a yeast two-hybrid screen as a binding partner for Retinoblastoma (Rb) (Li et al. 2000) and found to be a direct transcriptional target of Oct4/Sox2 in mouse embryonic stem cells using ChIP-PCR (Campbell et al. 2007). A recent report showed that FAM208A interacts with H3K9me3, a chromatin mark associated with gene silencing. Several other proteins encoded by the human homologues of genes detected in our screen were also identified as K9me3 interactors in this study (Eberl et al. 2013). The importance of these interactions is yet to be demonstrated in vivo. Using microarray analysis, two studies have reported that FAM208A expression levels are decreased in colorectal adenocarcinomas compared to normal colorectal tissue; however, it remains unclear whether these changes are a cause or consequence of tumourigenesis (Kucherlapati 2012; Seshagiri et al. 2012). Additional experiments using mouse mutants would be beneficial in clarifying the role of *D14Abb1e* in these settings.

## Conclusion

Our studies have revealed that *D14Abb1e* encodes a nuclear protein, which is critical for normal development in the mouse. The mutant mouse lines described here will be useful in future molecular studies to uncover the mechanism of action of *D14Abb1e* and its role in development and disease.
